# Accuracy of automatic deformable structure propagation for high-field MRI guided prostate radiotherapy

**DOI:** 10.1186/s13014-020-1482-y

**Published:** 2020-02-07

**Authors:** Rasmus Lübeck Christiansen, Lars Dysager, Anders Smedegaard Bertelsen, Olfred Hansen, Carsten Brink, Uffe Bernchou

**Affiliations:** 1 Department of Clinical Research, University of Southern Denmark, Winsløwparken 19 3. Sal, 5000 Odense C, Denmark; 2 Laboratory of Radiation Physics, Department of Oncology, Odense University Hospital, Kløvervænget 19, Indgang 85, Pavillion, Stuen, 5000 Odense C, Denmark; 3 Department of Oncology, Odense University Hospital, Kløvervænget 19 Indgang 85 Pavillion, 1. sal, 5000 Odense C, Denmark

**Keywords:** MR-linac, Radiotherapy, Prostate cancer, Computed tomography, Magnetic resonance, Deformable image registration, Treatment precision

## Abstract

**Background:**

In this study we have evaluated the accuracy of automatic, deformable structure propagation from planning CT and MR scans for daily online plan adaptation for MR linac (MRL) treatment, which is an important element to minimize re-planning time and reduce the risk of misrepresenting the target due to this time pressure.

**Methods:**

For 12 high-risk prostate cancer patients treated to the prostate and pelvic lymph nodes, target structures and organs at risk were delineated on both planning MR and CT scans and propagated using deformable registration to three T2 weighted MR scans acquired during the treatment course. Generated structures were evaluated against manual delineations on the repeated scans using intra-observer variation obtained on the planning MR as ground truth.

**Results:**

MR-to-MR propagated structures had significant less median surface distance and larger Dice similarity index compared to CT-MR propagation. The MR-MR propagation uncertainty was similar in magnitude to the intra-observer variation. Visual inspection of the deformed structures revealed that small anatomical differences between organs in source and destination image sets were generally well accounted for while large differences were not.

**Conclusion:**

Both CT and MR based propagations require manual editing, but the current results show that MR-to-MR propagated structures require fewer corrections for high risk prostate cancer patients treated at a high-field MRL.

## Background

Changes in anatomy over a radiotherapy (RT) treatment course for pelvic cancer has motivated adaptive treatment schemes [1, 2]. However, until now the limitations of cone beam CT (CBCT) image quality and soft tissue contrast has hampered the clinical implementation [3, 4]. With the introduction of magnetic resonance (MR) radiotherapy delivery systems, daily, MR guided adaptive radiotherapy (ART) has become possible with the potential to reduce the safety margins used today [5–7] and thus the treatment toxicity [8]. However, the workflow for an MR guided treatment fraction lasts longer than standard CBCT based linac treatment workflow, which increases the risk of patient motion during the treatment session [9–11]. One of the most time consuming steps in the MR workflow is the re-delineation and validation of target structures and organs at risk (OAR) [10, 12]. Automatic propagation of structures might also reduce the risk of misdelineations that could introduce systematic or large random errors. Two commercial MR linac (MRL) systems are currently clinically available and for both systems a deformable image registration (DIR) algorithm is part of the treatment planning system (TPS) to facilitate a fast deformation of the planning images and structure propagation to reduce this time.

The standard workflow suggested by the vendor for the high field MRL has been specified as propagation of structures from the planning CT scan (pCT) to the MR scan of the given treatment session. Previously, different commercial algorithms have been evaluated on different anatomies when performing DIR from CT to MR, showing great potential, but also uncertainties in the deformations [13–16]. Hence, manual revision and some manual corrections of the propagated structures would usually be required across the different commercial DIR solutions and anatomies [13, 17–19]. Therefore use of MR-MR DIR in the online MRL treatment workflow, as described by Bertelsen et al. [11], could provide more precisely propagated structures. However, to our knowledge, this has not yet been verified.

It has been demonstrated that manual delineation of soft tissue structures is more consistent when using MR rather than CT, both in terms of the inter- and intra-observer variation [20–22]. E.g. Smith et al. showed that the inter-observer variation of prostate on T2 weighted (T2w) MRI was smaller than on CT [20]. Furthermore, the volume of the prostate delineated on CT was larger than on MR [20, 22]. For these reasons the intra-observer variation on MR has been regarded as the reference precision in clinical practice [20, 21, 23].

This study investigates the geometric accuracy of deformable image registration of target volumes and OAR in high-risk prostate cancer patients for both CT-MR and MR-MR registrations using manual delineations as ground truth.

## Methods

### Patients

Twelve high-risk prostate cancer patients referred for 78 Gy for the prostate and proximal part of the seminal vesicles (SV) and 56 Gy for the pelvic lymph nodes, both delivered in 39 fractions on standard CBCT linacs, were included in the study. Exclusion criteria were contraindications to MR and metal implants in the pelvis (e. g. prosthetic hips).

The study has been approved by the regional board of ethics and all included patients have given their signed consent to participate.

### Image acquisition

Planning CT scans were acquired on either a Philips Big Bore Brilliance 16 slice scanner (Philips Medical Systems BV, The Netherlands) or a Toshiba Aquillion One (Canon Medical Systems Corporation, Japan). The scan parameters applied were: 50 cm field of view (FOV), 512 × 512 matrix and 3 mm slice thickness.

All MR scans were performed in treatment position on a Philips Ingenia 1.5 T (Philips Medical Systems BV, The Netherlands) equipped with a flat table top and fixation device for feet and knees. Supplementary to a planning MR (pMR) acquired immediately before or after the pCT, three sets of MRI (MR_10_, MR_20_ and MR_30_) were acquired at the 10th, 20th, and 30th fraction (allowing a variation of +/− 2 days) as representative samples of daily MRI sessions over their treatment course. The T2w sequence applied in this study was a 3D scan with 3D image distortion correction applied. The following parameters were used to resemble the sequence suggested for use at the MRL by the vendor: 300 slices, 400 × 400 mm FOV, 0.5 × 0.5 mm pixels, 1 mm slice thickness, TE 216 ms, TR 1800 ms. Scan time was 5.56 min.

The geometric fidelity of the MR scanner was evaluated weekly according to a quality assurance program including scans and analysis of a vendor specific phantom. MR sequences applied clinically were evaluated with a MagPhan RT 820 phantom (The Phantom Laboratory, Salem, NY, USA) scan analysed in Total QA software (Image Owl, Greewich, NY, USA).

### Structure delineation

For the current study, target volumes (prostate, SV and CTV56) were delineated on pCT and each acquired T2w MR set (pMR, MR_10_, MR_20_ and MR_30_) by one experienced oncologist. The prostate structure included the connective tissue capsule surrounding the gland. The elective volume CTV56 was defined as pelvic lymph nodes with a 7 mm margin as described by RTOG consensus guidelines [24]. All OAR (rectum, bladder, penile bulb, bowel and bilateral femoral heads) were delineated on each image set by one experienced RTT trained in delineation. For delineations on pCT a standard abdominal window/level settings were used (w 350/ l 40). Individual window/level settings were used for MRI delineations. Previous image sets were available for the observer during contouring, as they would be in a clinical setting.

These manual delineations represent the ground truth as to what each structure should encompass. The current study focuses on differences due to use of different image modality and not inter-observer variations. However the observed uncertainties are compared to the intra-observer variation which is less than the inter-observer variation. Thus, structures were re-delineated on all patients’ pMR by the same observers no less than one month after initial delineation to determine the intra-observer variation. In lack of the absolute truth, the intra observer-variation represents the best accuracy we can expect from the propagated structures [18].

Delineations were performed in the treatment planning system (TPS) dedicated to the Elekta Unity high-field MRL, Monaco ver. 5.40 (Elekta AB, Stockholm, Sweden). In this TPS, images are displayed using pixel interpolation.

### Image registration

**Fig. 1:**
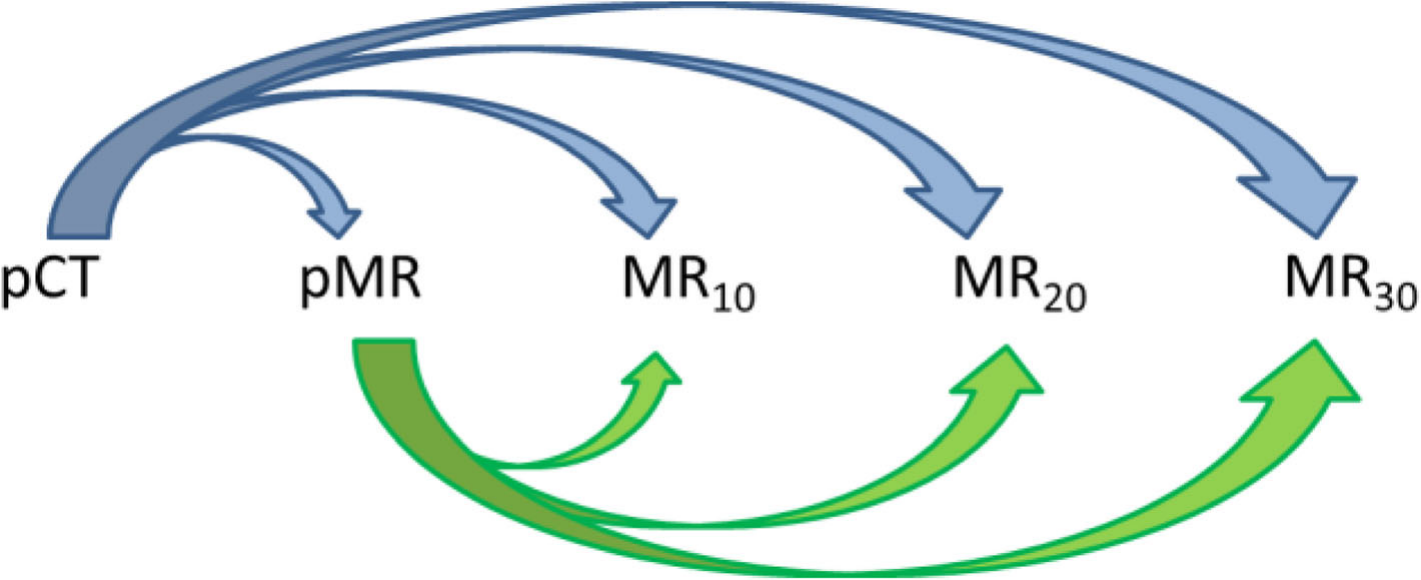
Schematic representation of image deformation and subsequent structure propagation from planning scans performed in the TPS

All registrations and structure propagations were performed in Monaco. The pCT was registered to each MR set and the pMR to each additional MR, as illustrated schematically in Fig. 1.

Standard settings of the commercial deformation software were used in all cases, specific details of the algorithm is not disclosed by the vendor. Deformation of CT images to MR was performed using normalized mutual information, whereas MR-MR deformations were performed using a local cross correlation algorithm.

### Structure comparison

**Fig. 2:**
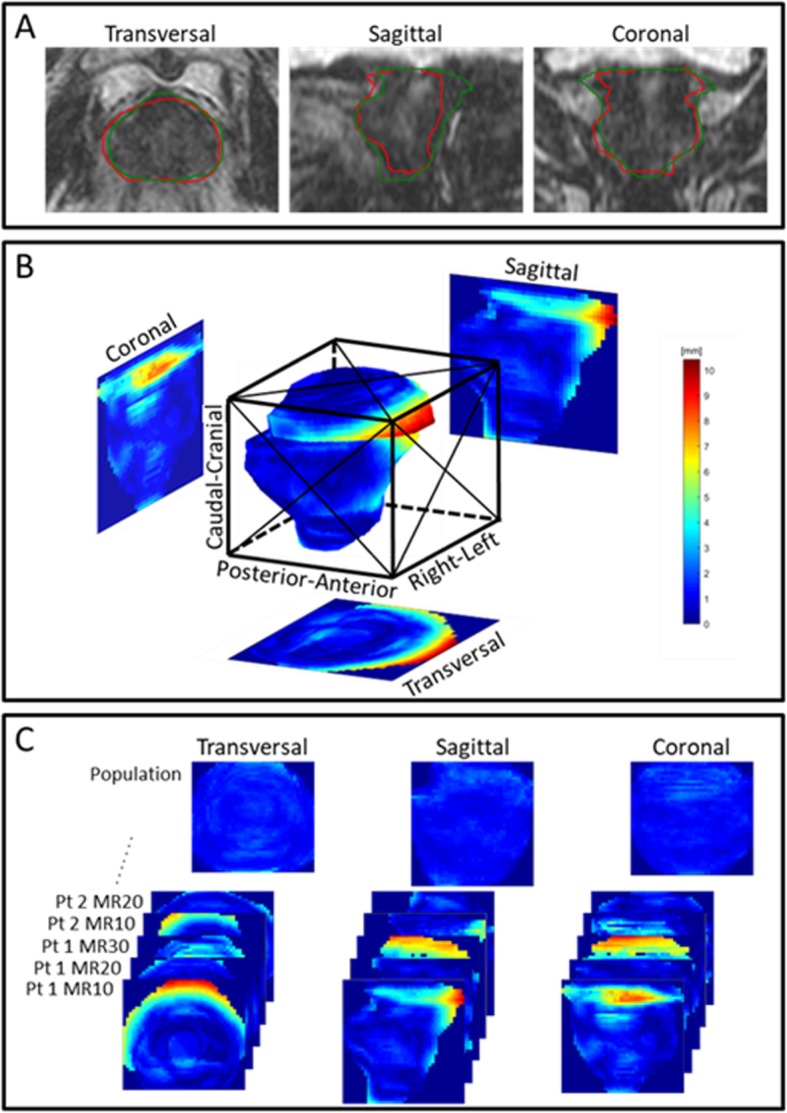
The manually delineated prostate (green) is defined as reference to the deformed prostate (red) are shown in **a**. Distance between the two is calculated in **b**, and projections onto the coronal, sagittal and transversal planes are made. In **c**, the projections are per projection summed over the patients to provide the population percentile surface distance projection image

Three metrics were calculated to evaluate the accuracy of DIR: The Dice similarity coefficient (DSC), which delivers the ratio of overlap between the manually delineated structure and the corresponding deformable propagated structure [25]. This measure is most relevant for smaller structures, as the index for large structures might be very high, although large clinically relevant volumes might not overlap. The mean surface distance (MSD), as described by Zukauskaite et al. [26] gives the average distance between the manual and deformed structure in absolute measures, which is particularly relevant for larger structures. The Hausdorff distance (HD) delivers the greatest distance between a given pair of structures to show a worst case scenario [27], thus very sensitive to outliers in the data. For each patient the average value over all the scans of the DSC, MSD and HD was calculated for each structure investigated for both MR-MR and CT-MR registrations and compared to the intra-observer variation. In order to assess spatial patterns in the distribution of variation between ground truth and deformed or re-delineated contours, population based surface distance projection images were generated in the transversal, sagittal, and coronal plane for each structure. These projection images, showing the differences between ground truth and deformed structures or intra-observer variation, were created using the following procedure (see Fig. 2 for a graphical overview): The smallest possible bounding box, oriented along the main patient directions (Anterior-Posterior, Right-Left, and Cranio-Caudal), surrounding a given patient organ was initially defined. For all voxels at the surface of the organ, the distance to the reference organ was measured and then projected into the sagittal, transversal, and coronal plane. In these three planes, a grid encompassing the bounding box and with fixed number of pixels was defined (Additional file 1: Table A1). Within each pixel the projected deviations was averaged in order to observe scan specific spatial structures within the uncertainty. To provide the population based surface distance projection images, the 50 as well as the 90 percentile of all the scan individual projection deviations were subsequently found in each pixel. Although a specific type of structure may have varying size and shape depending on scan and patient, fixing the number of pixels in the three planes allow the generation of population based maps where the approximate distribution of distances, uncertainties can be assessed. The final projection images are shown with equal width and an aspect ratio corresponding to the mean structure based on all scans of all patients.

### Statistical analysis

Differences between patient median DSC, MSD and HD values between CT-MR, MR-MR registrations and ground truth, as well as difference in structure volume between pCT and pMR, were tested for statistical significance by Wilcoxon signed-rank tests at the 5% significance level.

## Results

**Fig. 3:**
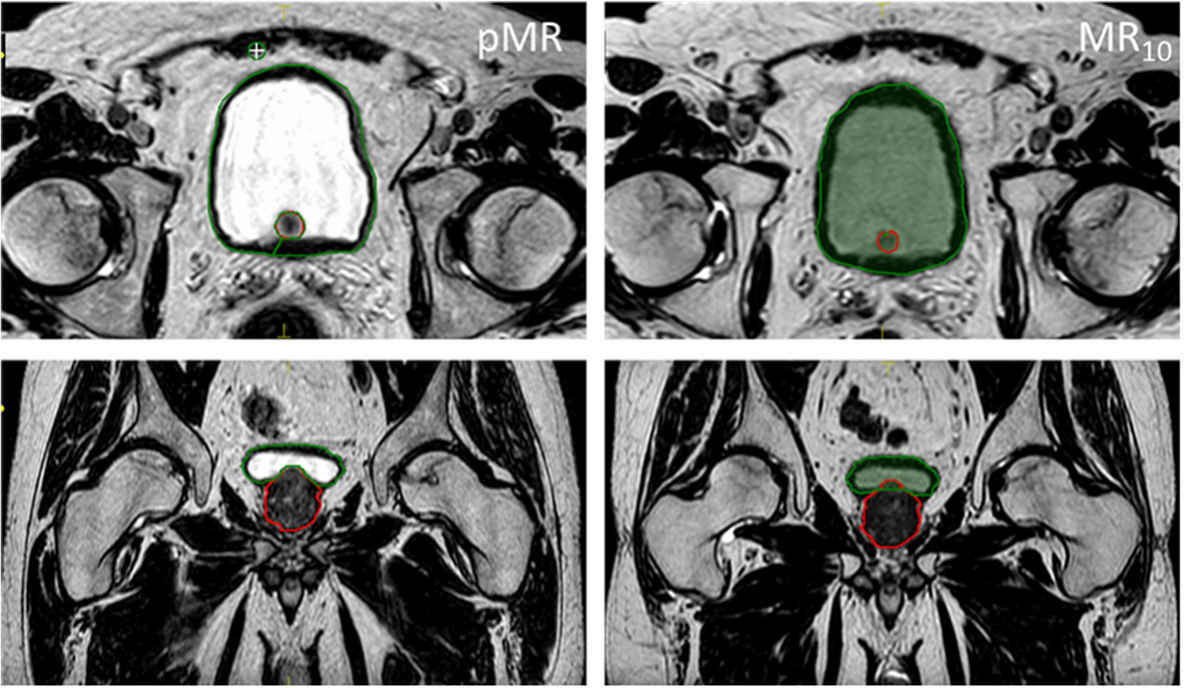
Prostate (red) and bladder (green) delineated manually on pMR, shown in transverse and coronal view on the left. Note that the bladder and prostate do not occupy the same space. On the right these structures have been propagated onto MR_10_, and the bladder structure overlaps the prostate

In total 672 structures were created successfully by DIR propagation. However, for 25% of the patients (3/12) the bladder was not propagated correctly because of the TPS’ inability to deform pin hole structures (the TPS’ best approximation of a donut structure) correctly, as illustrated in Fig. 3. All MR-MR propagated structures yielded higher population median DSC than CT-MR propagations when compared to ground truth delineations (Table 1 A), implying that MR-MR DIR is more accurate. This was statistically significant for the prostate, SV, rectum, femoral heads and penile bulb.

**Table 1:** Population median DSC (A), MSD (B) and HD (C) for all patients of the 4 CT-MR propagations, 3 MR-MR propagations, intra-observer variations (IOV) and their differences (Δ) are shown for each structure

A	Population median DSC								
				CT-MR vs MR-MR		CT-MR vs IOV		MR-MR vs IOV	
	CT-MR	MR-MR	IOV	Δ	*p*-value	Δ	*p*-value	Δ	*p*-value
Prostate	0.84	0.90	0.92	−0.07	< 0.001	− 0.08	< 0.001	− 0.02	0.01
SV	0.68	0.76	0.81	−0.08	0.001	−0.3	< 0.001	−0.05	0.05
CTV56	0.84	0.84	0.87	0.0	0.73	−0.03	0.02	−0.03	0.23
Rectum	0.77	0.87	0.95	−0.10	< 0.001	−0.18	< 0.001	− 0.08	0.003
Bladder	0.87	0.92	0.97	−0.05	0.15	−0.10	< 0.001	−0.05	< 0.001
R fem. Head	0.93	0.95	0.95	−0.02	< 0.001	−0.02	0.005	−0.00	0.18
L fem. Head	0.91	0.94	0.94	−0.03	0.007	−0.03	0.002	0.00	0.20
Penile Bulb	0.64	0.79	0.85	−0.15	< 0.001	− 0.21	< 0.001	− 0.06	0.08
B	Population median MSD [mm]								
				CT-MR vs MR-MR		CT-MR vs IOV		MR-MR vs IOV	
	CT-MR	MR-MR	IOV	Δ	*p*-value	Δ	*p*-value	Δ	*p*-value
Prostate	1.60	1.00	0.88	0.60	< 0.001	0.72	< 0.001	0.12	0.15
SV	1.48	1.17	0.86	0.31	0.002	0.62	0.005	0.31	0.13
CTV56	1.99	1.82	1.58	0.17	0.30	0.41	< 0.001	0.24	0.13
Rectum	2.41	1.25	0.65	1.16	0.007	1.76	< 0.001	0.6	0.001
Bladder	1.96	1.11	0.55	0.85	0.03	1.41	< 0.001	0.56	< 0.001
R fem. Head	1.09	0.81	0.75	0.28	0.002	0.34	0.005	0.06	0.34
L fem. Head	1.37	0.81	1.05	0.56	0.03	0.32	0.03	−0.24	0.42
Penile Bulb	1.61	0.89	0.74	0.72	< 0.001	0.87	0.002	0.15	0.13
C	Population median HD [mm]								
				CT-MR vs MR-MR		CT-MR vs IOV		MR-MR vs IOV	
	CT-MR	MR-MR	IOV	Δ	*p*-value	Δ	*p*-value	Δ	*p*-value
Prostate	7.16	5.10	4.89	2.16	0.005	2.06	< 0.001	2.27	0.42
SV	6.55	5.54	5.31	1.01	0.18	1.01	0.04	1.24	0.42
CTV56	14.92	12.77	11.55	2.15	0.34	2.15	0.03	3.37	0.42
Rectum	12.36	8.89	7.65	3.47	0.38	3.47	0.06	4.71	0.47
Bladder	10.88	5.71	4.05	5.17	0.38	5.17	< 0.001	6.83	0.01
R fem. Head	4.96	4.77	4.41	0.13	0.38	0.19	0.42	0.55	0.62
L fem. Head	4.98	4.75	5.21	0.23	0.30	0.23	0.85	−0.23	0.85
Penile Bulb	6.38	3.96	3.46	2.42	< 0.001	2.42	0.002	2.92	0.38

Statistical significance of differences is given by the *p*-value

An ideal DSC is 1, whereas ideal MSD and HD are 0

Population median MSD values demonstrated better agreement with the ground truth for all deformed structures based on MR relative to CT (MR-MR values were 0.17–1.16 mm lower). Differences were statistically significant for all structures except CTV56 (Table 1 B). Overall, CT-MR deformed structures showed inferior DSC and MSD compared to the intra-observer variation in contrast to MR-MR registration. MR-MR deformed structures generally showed population median DSC and MSD values in the same range as the intra-observer variations, but only statistically significant in prostate, bladder and rectum.

MR-MR DIR yielded smaller HD for all 8 investigated structures than CT-MR but were only statistically significant for prostate and penile bulb (Table 1 C).

Population median DSC and MSD showed statistically significant difference between CT-MR propagated contours and the intra-observer variation for all organs (Table 1 A). MR-MR was statistically similar to the intra observer variation in most cases (4 of 8 for DSC and 6 of 8 for MSD).

**Fig. 4:**
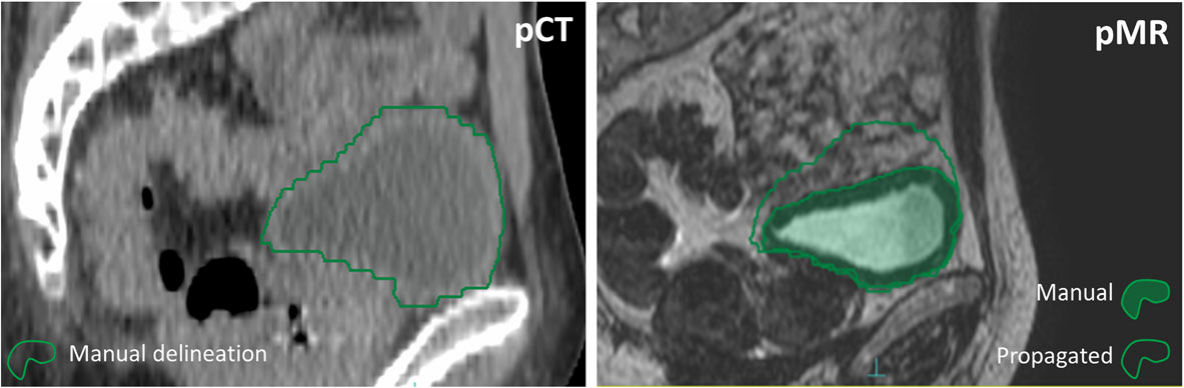
The large bladder volume seen on pCT and deformed to pMR matched the bladder seen on pMR poorly. Generally, large differences were not well accounted for by the deformable structure propagation

Visual inspection of the deformed structures and their source and destination image sets revealed that small differences between organs in the two image sets were generally well accounted for by the DIR algorithm. Contrarily, large differences were in most cases not at all compensated, as exemplified in Fig. 4. This effect impacted on the MSD and DSC values; deformable organs, such as the bladder and rectum, showed larger disagreements than rigid structures, e. g. the prostate and femoral heads. The difference in size between pCT or pMR and MR_x_ (Table 2) potentially affects the resulting deformation quality. Large variations in volume were observed for bladder and rectum delineations. The prostate structure was systematically smaller when delineated on MR than on CT (*p* <  0.001).

**Table 2:** Median volumes, and their range in brackets, of prostate, bladder and rectum structures manually delineated on planning images. Statistical test of the difference between volumes on CT versus MR was performed and the result given as the *p*-value

Structure	V (pCT) [cm^3^]	V (pMR) [cm^3^]	*p*-value
Prostate	45.5 (27.2–132,5)	38.1 (23.7–112.6)	< 0.001
Bladder	186.5 (85.8–460.5)	174.3 (57.7–534.4)	0.25
Rectum	96.1 (54.3–133.0)	94.0 (38.3–136.8)	0.16

**Fig. 5:**
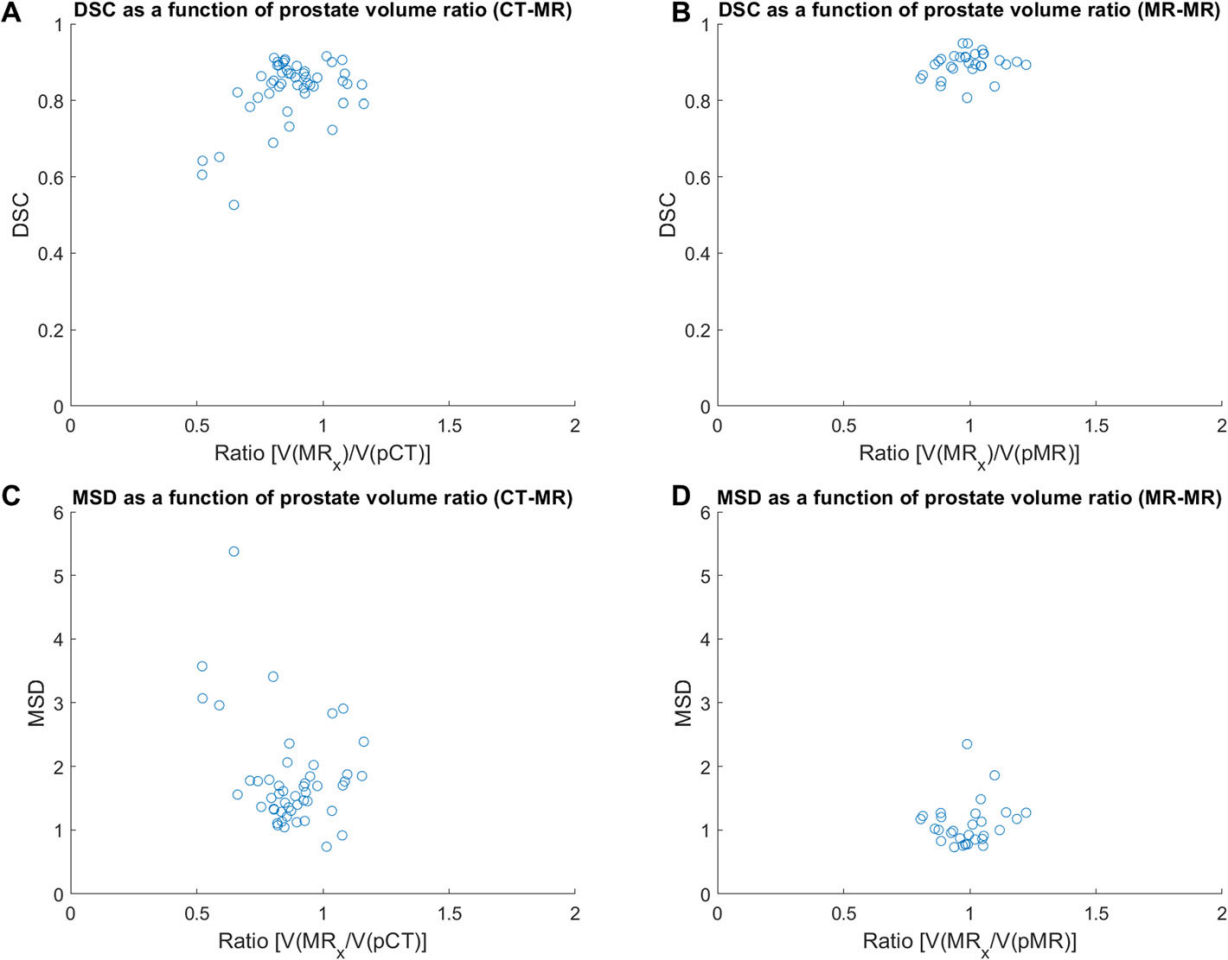
DSC and MSD plotted against the ratio of ground truth prostate volumes of planning images (pCT or pMR) and the image of the day (MR_x_)

In Fig. 5, the DSC and MSD were plotted against the ratio of structure volume on the planning image versus the ground truth on the daily image (MR_x_). For the prostate, only small deviations were observed between the volumes of the structure between the planning image and subsequent images, which translates to consistently high DSC and low MSD for all deformations. Still, a tendency of DSC being closer to 1 and low MSD values for ratios near 1 was observed.

**Fig. 6:**
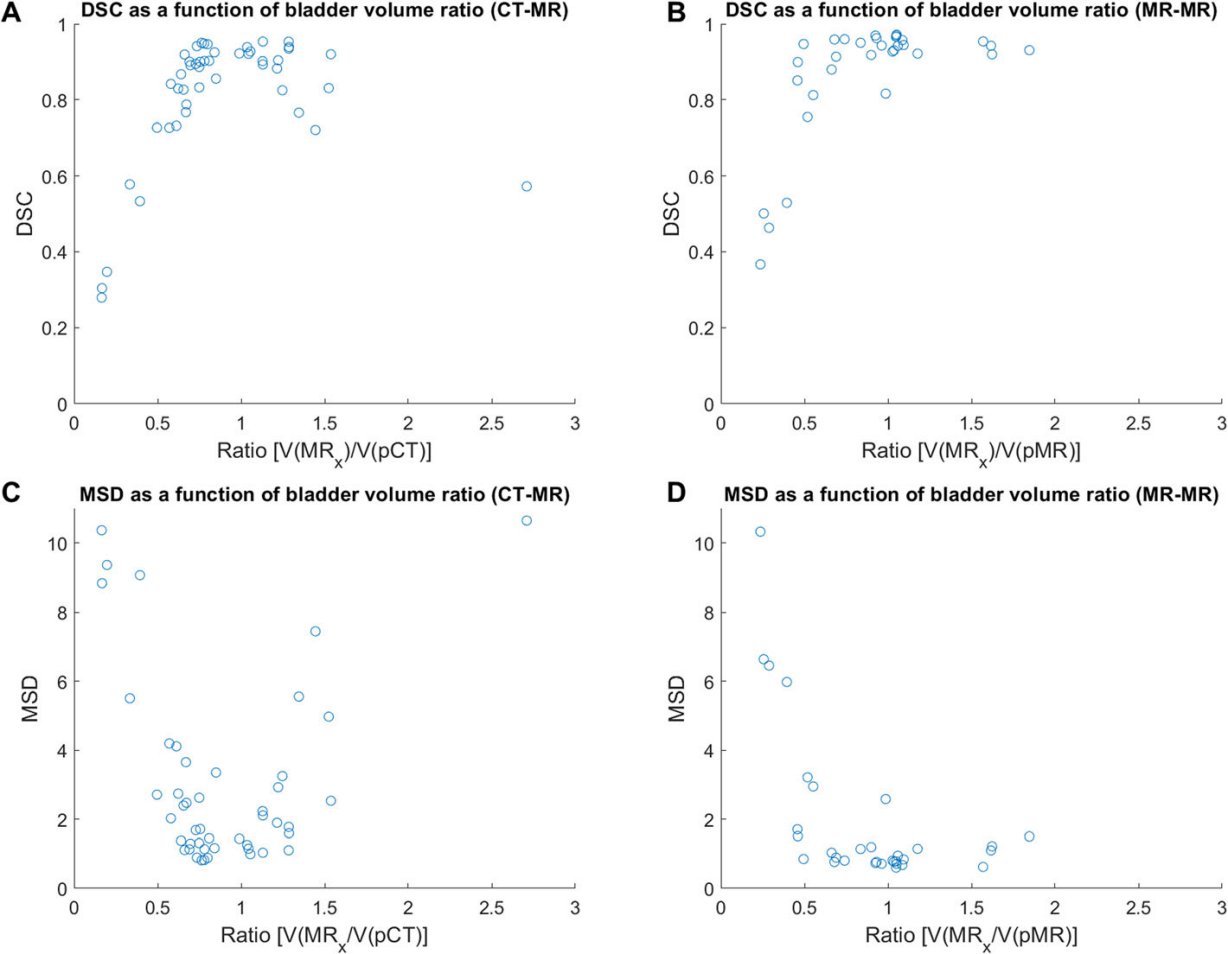
DSC and MSD plotted against the ratio of ground truth bladder volumes of planning images (pCT or pMR) and the image of the day (MR_x_)

A correlation between volume ratio and accuracy of propagated structures was seen for the bladder by the parabolic tendency in the plots with extrema approximately at volume ratio 1 (Fig. 6). A similar pattern was seen for rectum propagations, although less pronounced than for the bladder (Additional file 1: Figure A1).

**Fig. 7:**
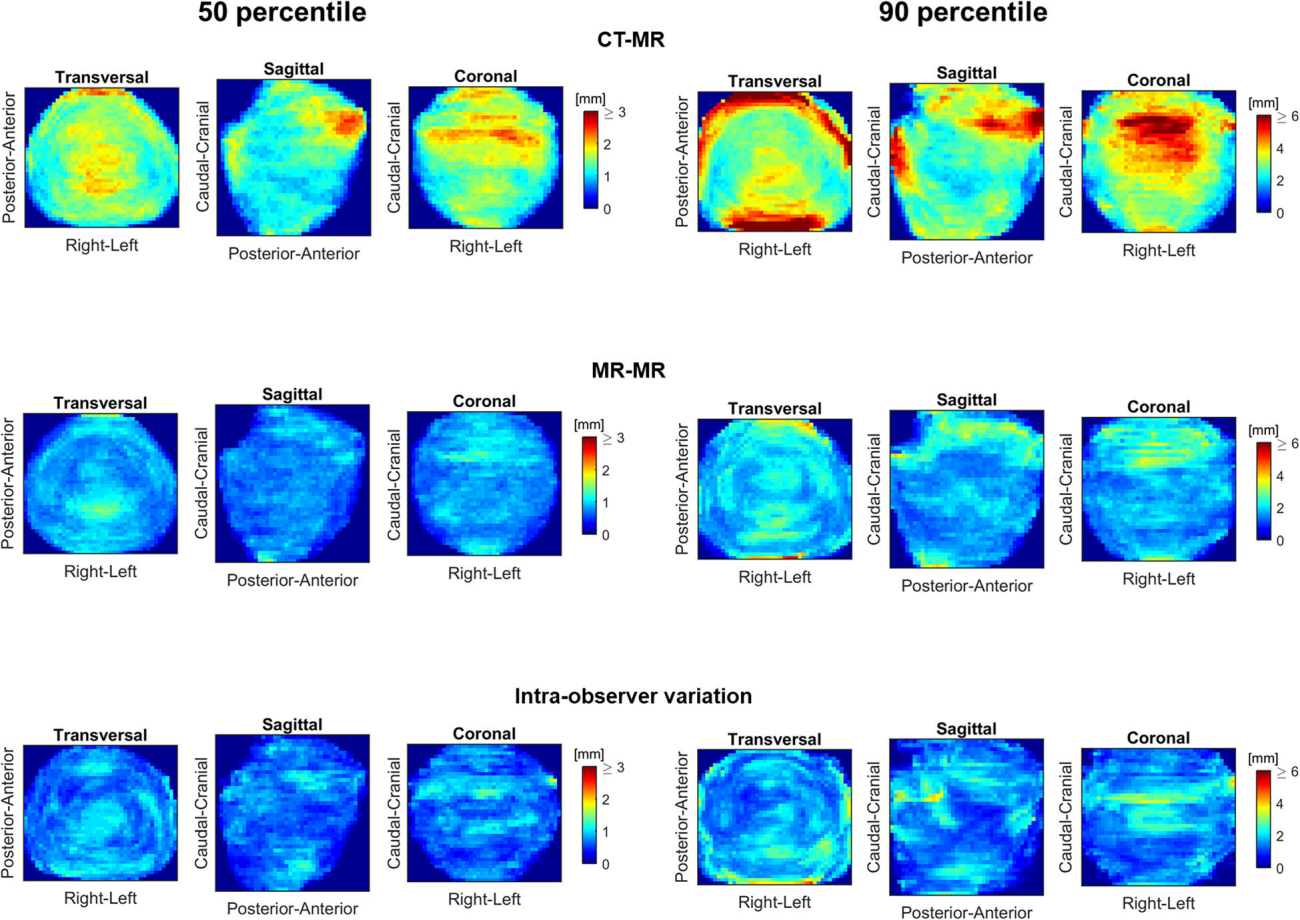
50 and 90 percentile surface distance projection images for the prostate based on CT-MR and MR-MR registrations as well as the intra-observer variation

The population difference between ground truth and deformed or re-delineated structures are visualized for the prostate in Fig. 7. The projection images show that the delineation differences overall were larger for CT-MR compared to MR-MR. Thus, the observed difference between the image modalities was not only related to specific volumes of the prostate, but also variation in delineations due to different image modalities. The largest error in DIR propagated prostate structures were seen in the anterior-cranial part of the gland and towards the rectum for CT-MR deformations (Fig. 7).

**Fig. 8:**
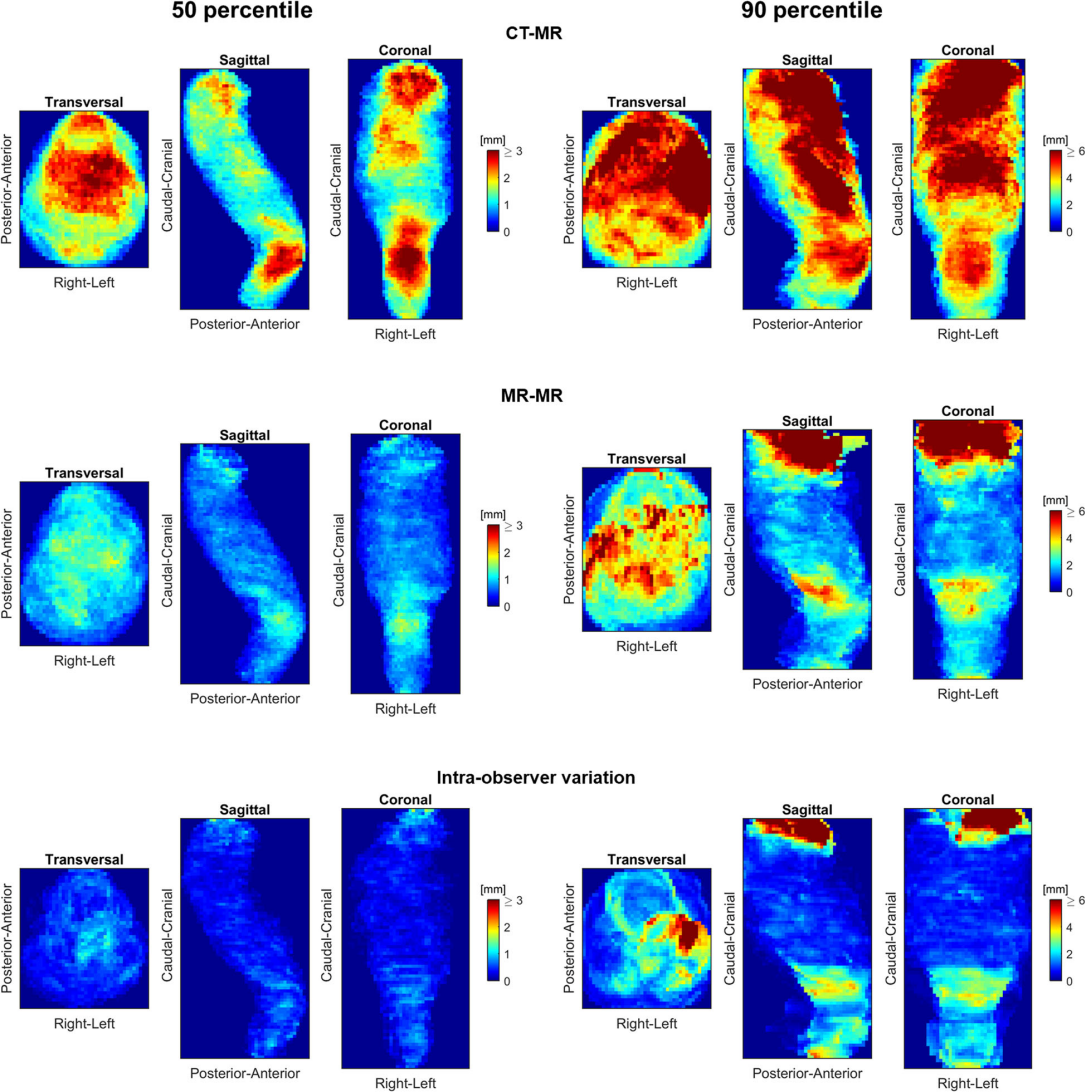
50 and 90 percentile surface distance projection images for the rectum based on CT-MR and MR-MR registrations as well as the intra-observer variation

A similar pattern was observed in the population projection images for rectum (Fig. 8); overall, variations were larger for CT-based DIR with the largest differences towards the cranial boundary and the anterior wall. MR-MR DIR propagated structures were generally in good agreement, although cranially differences exceeded 3 mm. The trend was similar for the intra-observer variation. Projection images of the remaining investigated structures showed similar patterns; the largest deviations between both CT-MR and MR-MR propagated structures and ground truth was seen at the cranial and caudal limits of CTV56 (Additional file 1: Figure B1). The seminal vesicles showed the largest deviations anteriorly for CT-MR, but cranially for MR-MR (Additional file 1: Figure B2). The penile bulb propagations showed the largest deviations anteriorly for CT-MR, while MR-MR propagations were similar to the intra-observer variation (Additional file 1: Figure B6).

## Discussion

This validation study has shown that intra-modal MR-MR image deformation is almost comparable in accuracy to the intra-observer variation of manual delineations. Inter-modal CT-MR DIR was less accurate and thus not ideal for clinical use. CT-MR deformations were less accurate than the intra-observer variation and therefore will require more time for manual editing. This is problematic for online treatment adaptation due to the inconsistency in target and OAR definition but also due to the risk of internal organ motion increases. Because MR-MR deformations were similar in accuracy to intra-observer variations and thus require less manual corrections, the delineating clinician might have a better starting point when defining the target and OAR, and the risk of errors in delineation decreases.

This result is similar to what has been shown to be the case for manual delineations: MR provides higher consistency of the delineations. In this light, the workflow using the pCT as the source for DIR to the session MR, as proposed as standard by the MRL vendor, might be suboptimal. Instead, a workflow using the pMR as the source would provide a set of propagated contours that better represent their actual shape and position. Hence, time required to review and revise while the patient is on the couch, is minimized. From the population surface distance projection images, it appears that some DIR propagation errors are systematic: Both for CT-MR and MR-MR the prostate differs mainly in the most anterior and cranial region, CTV56 at the caudal and cranial boundaries. Variations in bladder and rectum filling require corrections cranially and the penile bulb generally requires corrections anteriorly. Comparison of the 90 percentile surface distance projection images for MR-MR to the related intra-observer variation reveals that regions of large DIR uncertainty are also regions of large intra-observer variation, thus probably regions for which it inherently is difficult to define the “ground truth” of the delineation. Similar observations were made for rectum (Fig. 5), CTV56 (Additional file 1: Figure B1) and penile bulb (Additional file 1: Figure B6). Since some structures appear more difficult to deform accurately for the DIR algorithm, it might produce better results if future algorithms include organ specific information, thereby allowing, e. g., larger variations in the cranio-caudal direction for the bladder. However, with the currently available DIR algorithm, the correlation found between large differences in the bladder volume on the planning image and daily image, indicates that minimizing the variation between the source image and images of the day may be appropriate, e. g. by using a drinking protocol. The current results indicates that only for volume ratios in the range of 0.8–1.2 result in reasonably accurate DIR structure propagations. Neither the CT-MR nor the MR-MR DIR was able to account for large volume changes.

It should be noted that in this study, the slice thickness of CT (3 mm) and MR (1 mm) differ. This means that by default there can be a difference between delineations cranially and caudally of up to 2 mm due to this fact alone. For structures of little extension in the caudo-cranial direction, but large in other directions this could have a non-negligible impact, favoring MR-MR DIR in this study. Equally, the difference in the pixel size between CT and MR could mean differences of up to 0.5 mm right–left and anterior-posterior directions. However, the population surface distance projection images of this study do not indicate that the difference in slice thickness severely impacts these results, as the geometric distribution of differences is the same for CT-MR as MR-MR. E.g. the difference between ground truth and DIR propagated contours is not increased in the most cranial and caudal regions of rigid structures such as the femoral heads for the CT-MR registrations compared to the more central regions (see Figure B4 and B5 in the Additional file 1).

Image quality itself could affect image registration and resulting structure propagations. In the current study, MR image acquisition during the course of treatment was made on a diagnostic MR scanner, rather than on the MR Linac itself. Although the scan sequence used were set up as closely as possible to the proposed clinical settings on the MRL, the acquired image quality is not identical to that of the MRL, as much of the hardware is designed differently, e. g. the magnet, gradient magnets and coils. A detailed comparison of image quality between the two scanners is beyond the scope of the current study. However, it has previously been demonstrated that the geometric fidelity of the scanners are similar [28].

For a workflow using MR as the primary image set for dose planning, electron densities required for dose calculations must be generated either based on a pCT or alternatively from a MR based pseudo CT in order to apply a MR-only planning workflow [29–31]. ART can also be performed using CBCT, which does provide ED information, which, with correct adjustments, can provide the basis for precise dose calculations [32–34]. With current standard CBCT technology, the accuracy of CT DIR to CBCT is similar to that of the inter-observer variation [35, 36]. Future publications will document if new CBCT based with iterative image reconstruction technologies are able to increase deformation accuracy.

The organs at risk evaluated in this study are relevant for other pelvic indications than prostate cancer. Further studies will have to assess whether the DIR accuracy observed here also apply in other anatomical regions. Systematic shrinkage or swelling of organs is not expected for prostate cancer patients over the course of RT [37]. Therefore, continuously using the pCT and pMR, rather than successively using the latest acquired MR, should not affect the resulting overall accuracy of the DIR. In other indications, e.g. head and neck cancers and lung cancers, tumor shrinkage has been observed [38, 39]. For such indications, it might be appropriate to successively use the last acquired MR for DIR for daily plan adaptation.

## Conclusion

This study has shown that for high risk prostate cancer patients to be treated with an adapted plan on the Unity MRL, structures propagated from planning scan images to online daily MR images need manual editing, whether they were propagated from CT or MR. But the current results show that MR-MR propagated structures require fewer corrections and are therefore preferred for clinical use, as the online planning time may decrease and delineation accuracy increase compared to a CT-MR workflow. Thus, a clinical MR-MR workflow has been implemented locally.

## Supplementary information

**Additional file 1.** High resolution versions of images in the appendices are provided in the following files.

## Data Availability

Please contact author for data requests.

## Abbreviations

ART: Adaptive radiotherapy
CBCT: Cone beam computed tomography
CT: Computed tomography
CTV: Clinical target volume
DIR: Deformable image registration
DSC: Dice similarity coefficient
FOV: Field of view
HD: Hausdorff distance
IOV: Intra-observer variation
L: Left
MR: Magnetic resonance
MRI: Magnetic resonance imaging
MRL: Magnetic resonance linac
MSD: Mean surface distance
OAR: Organ at risk
pCT: Planning computed tomography
pMR: Planning magnetic resonance
R: Right
RT: Radiotherapy
RTT: Radiation therapist
SV: Seminal vesicles
TE: Time to echo
TPS: Treatment planning system
TR: Time to repeat
VMAT: Volumetric modulated arc therapy
