# Supplementary material for: Accuracy of automatic deformable structure propagation for high-field MRI guided prostate radiotherapy
Source: Radiat Oncol. 2020 Feb 7;15:32. doi: 10.1186/s13014-020-1482-y (PMC7007657; doi:10.1186/s13014-020-1482-y)
Supplement: Supplementary file 1 — Additional file 1. High resolution versions of images in the appendices are provided in the following files. [file 13014_2020_1482_MOESM1_ESM.docx]

## Additional material

## Appendix A

| Number of pixels | | | |
| --- | --- | --- | --- |
|  | Left-right | Caudal-cranial | Anterior-posterior |
| Prostate | 40 | 40 | 40 |
| SV | 40 | 20 | 20 |
| CTV56 | 80 | 80 | 80 |
| Rectum | 40 | 80 | 40 |
| Bladder | 80 | 40 | 80 |
| R Femoral Head | 40 | 40 | 40 |
| L Femoral Head | 40 | 40 | 40 |
| Penile Bulb | 20 | 20 | 20 |

*Table A1. Number of pixels used to generate population percentile surface distance projection images.*

**
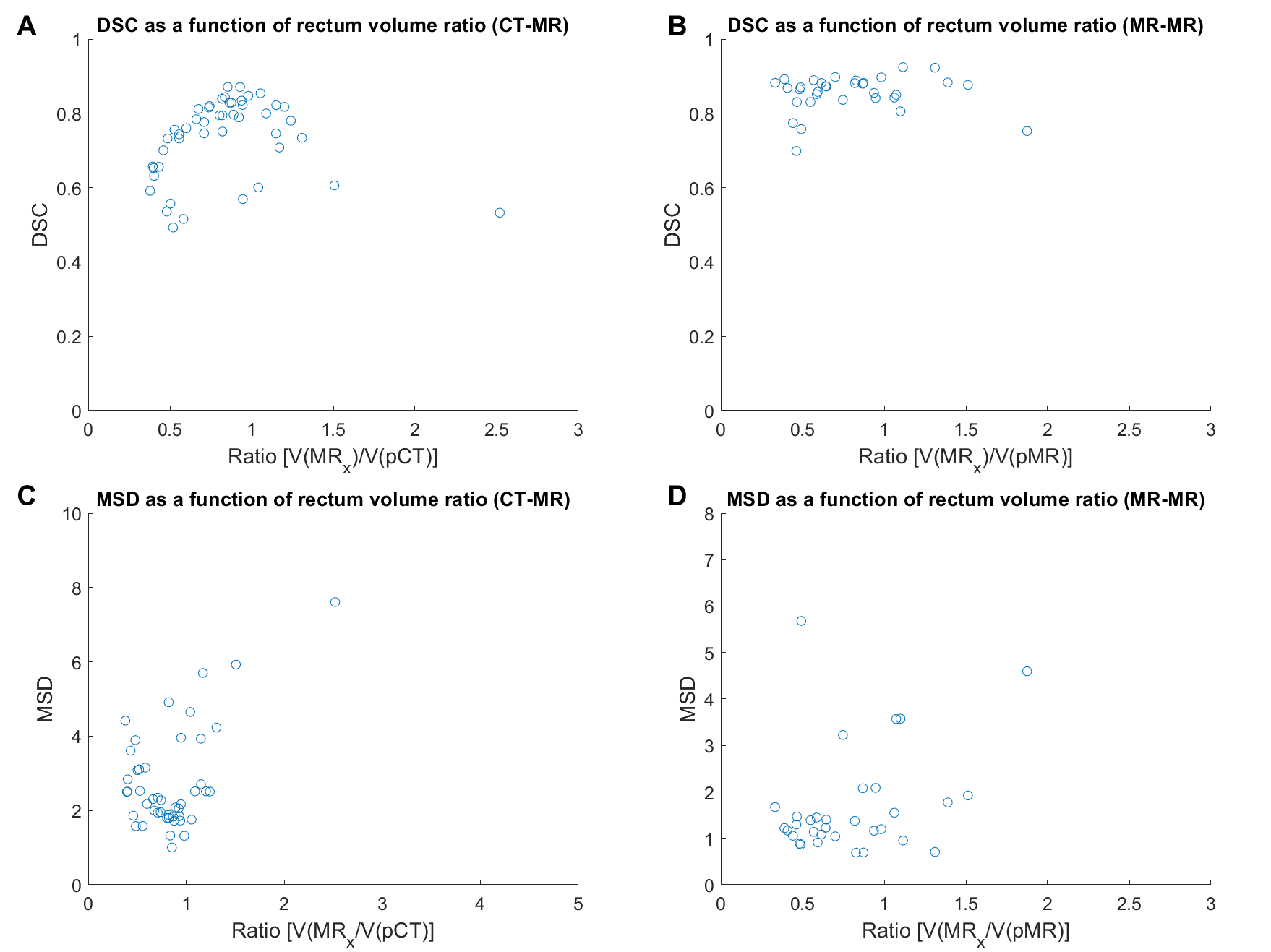
**

*Figure A1. DSC and MSD plotted against the ratio of ground truth rectum volumes of planning images and the image of the day.*

## Appendix B

**CTV56 projection image**


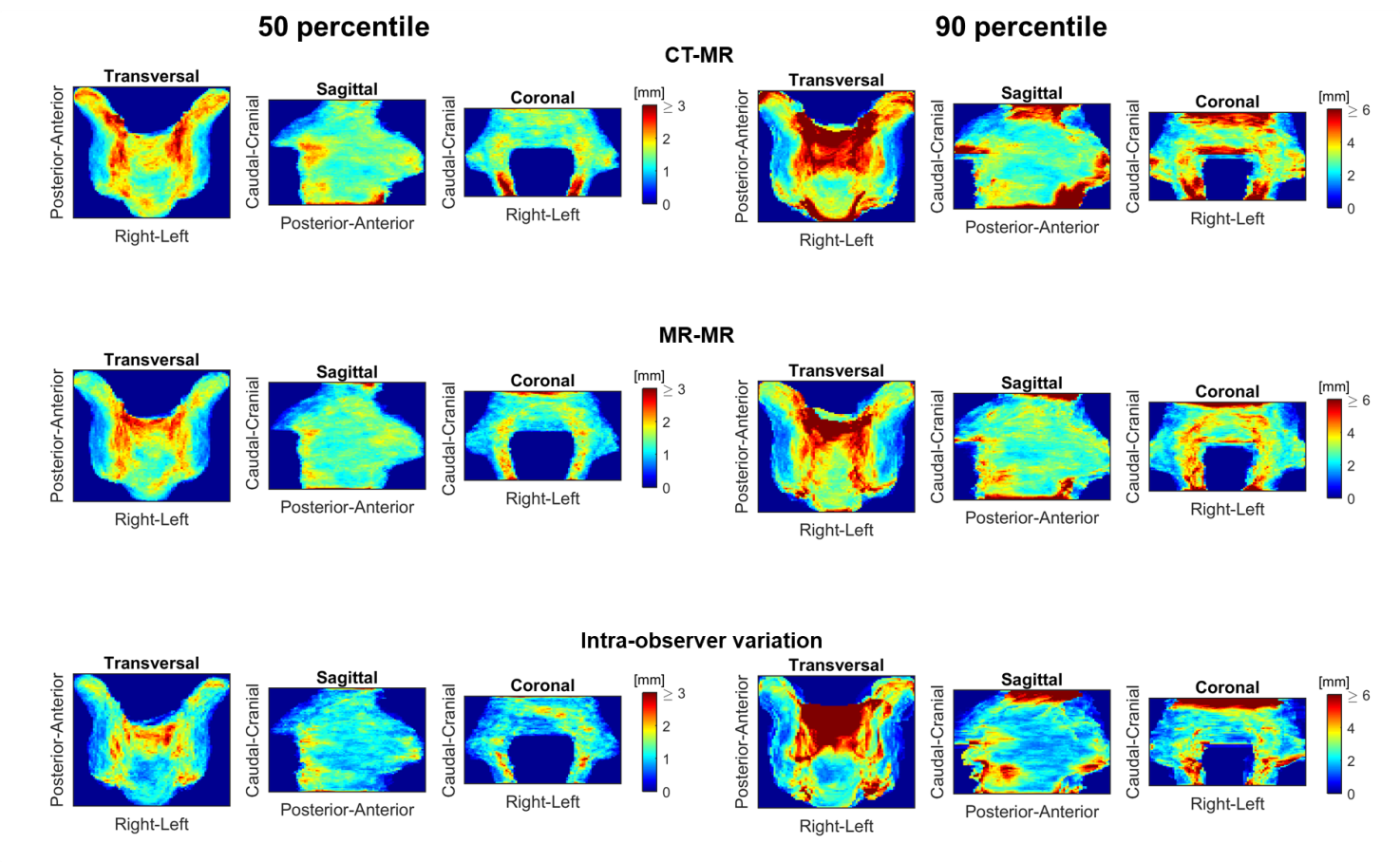


*Figure B1. 50 and 90 percentile surface distance projection images for CTV56 based on CT-MR and MR-MR registrations as well as the intra-observer variation.*

**Seminal vesicles projection image**


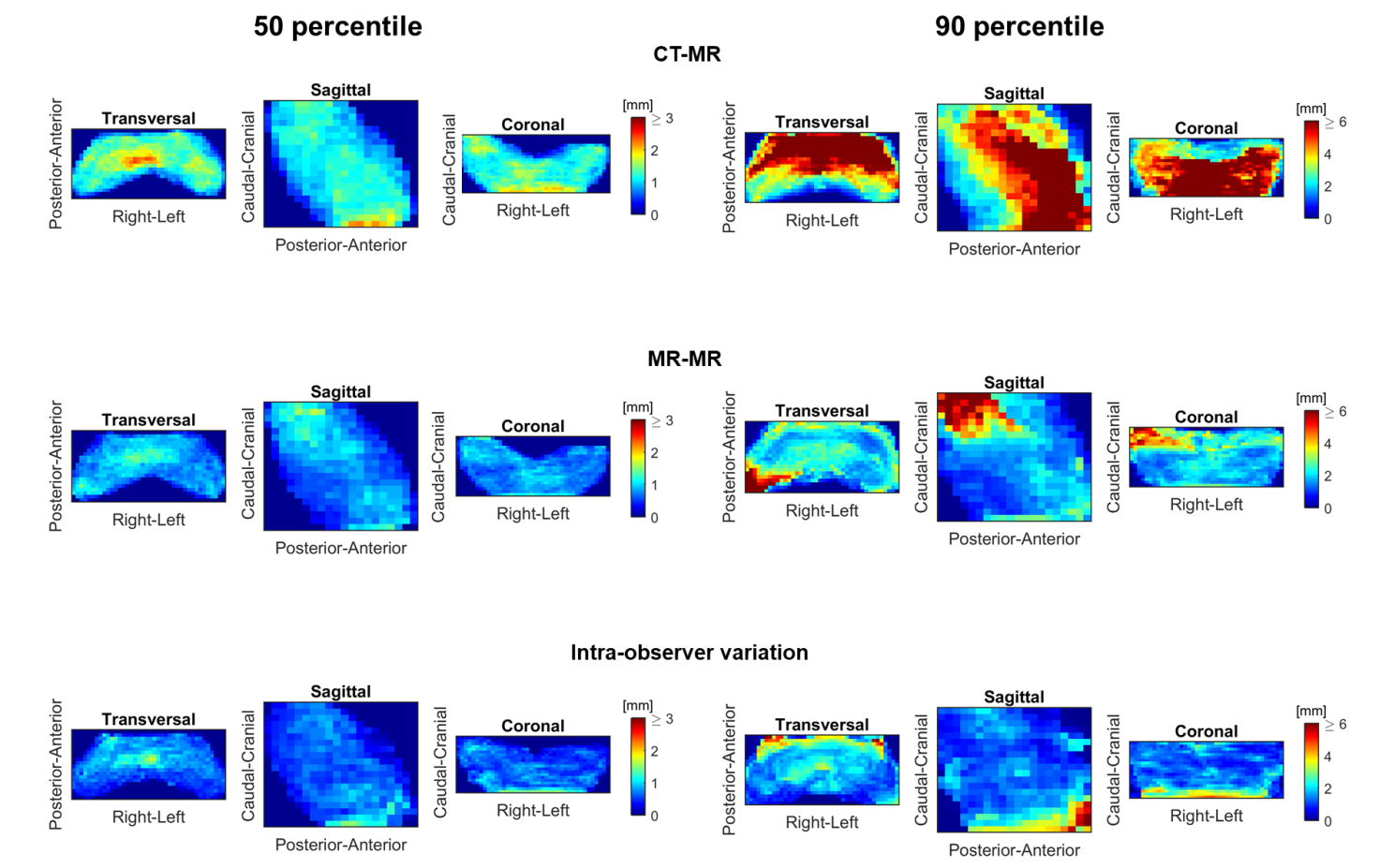


*Figure B2.* *50 and 90 percentile surface distance projection images for SV based on CT-MR and MR-MR registrations as well as the intra-observer variation.*

**Bladder projection image**


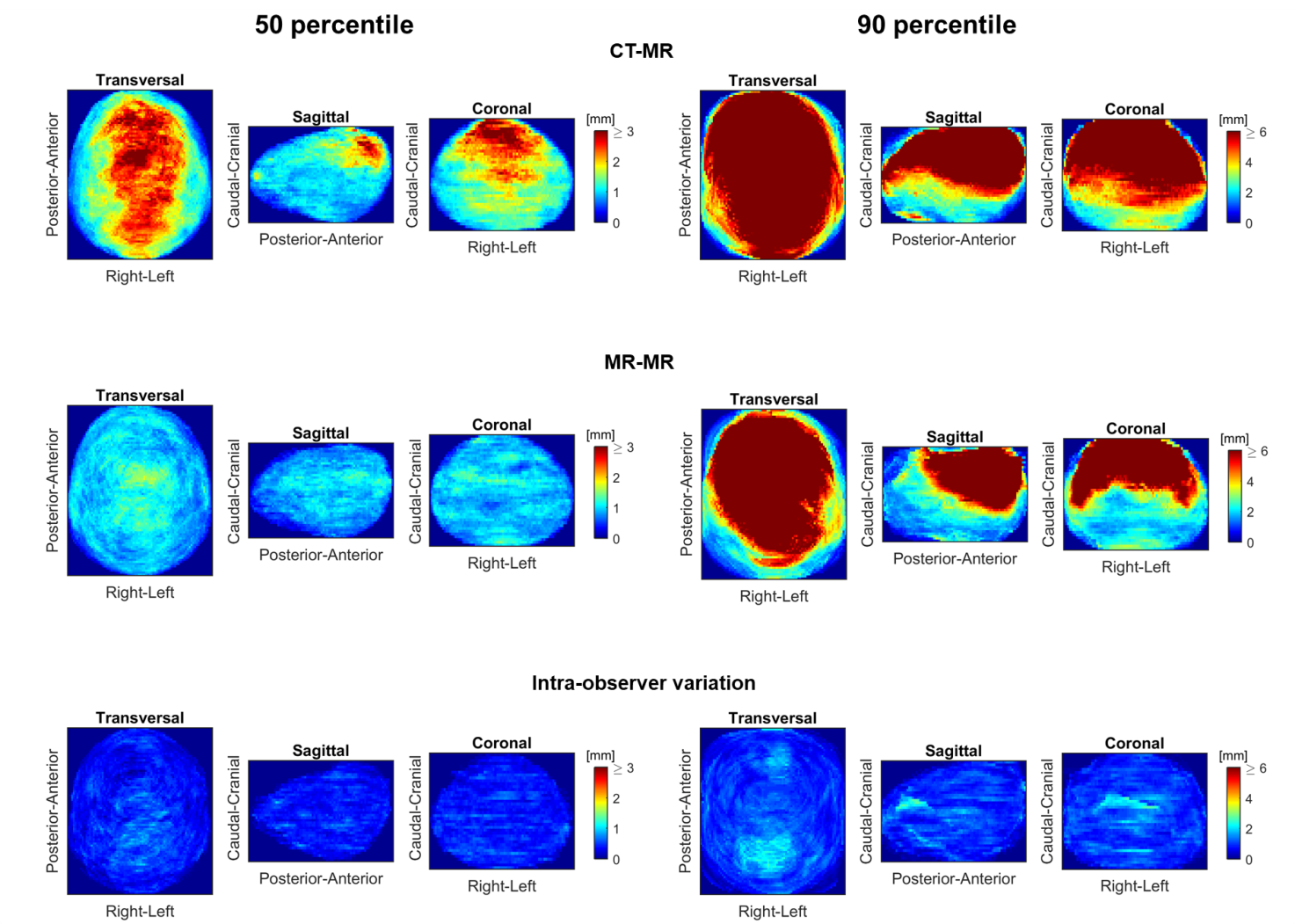


*Figure B3.* *50 and 90 percentile surface distance projection images for bladder based on CT-MR and MR-MR registrations as well as the intra-observer variation.*

**Right femoral head projection image**

**
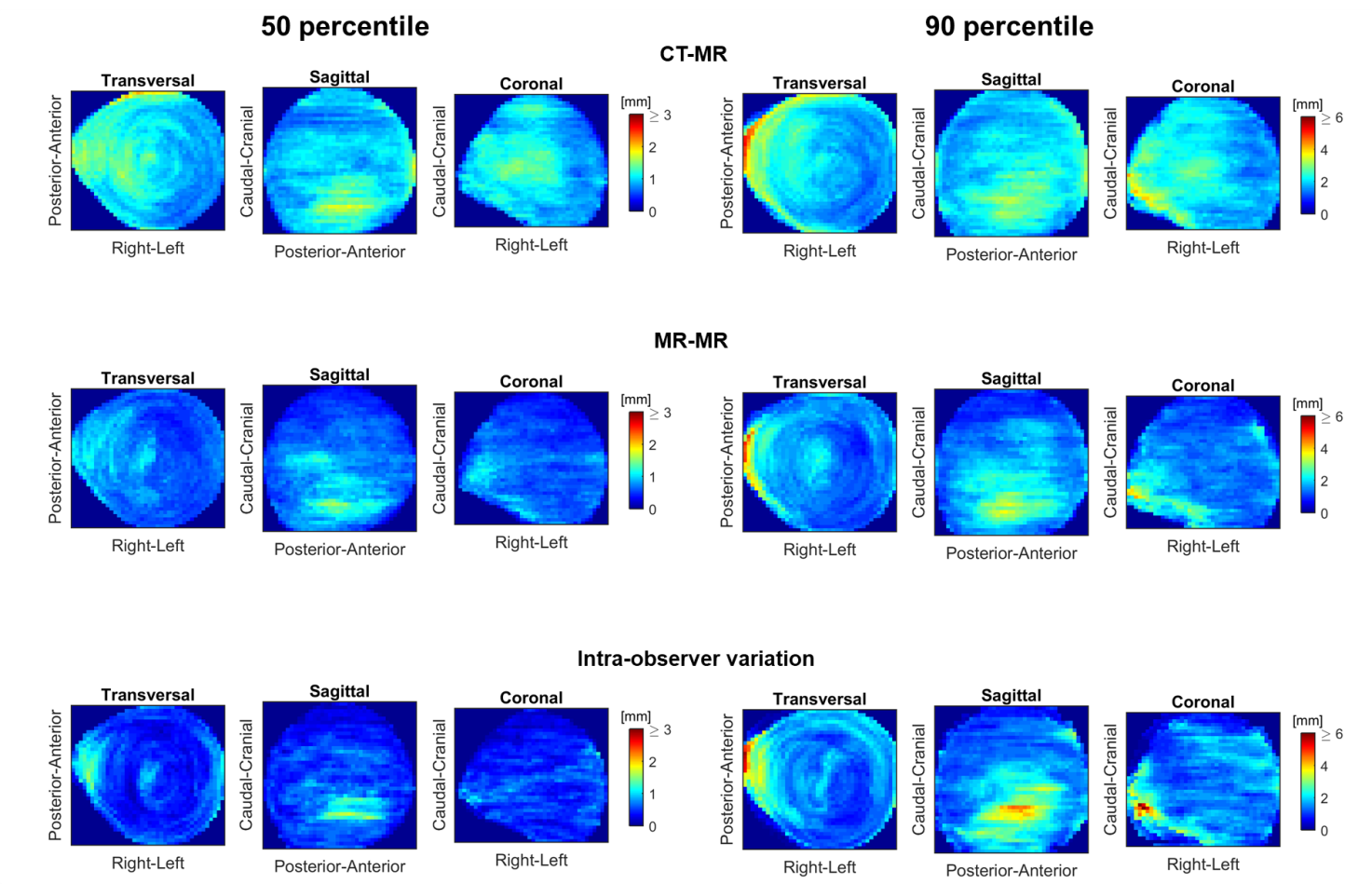
**

*Figure B4. 50 and 90 percentile surface distance projection images for right femoral head based on CT-MR and MR-MR registrations as well as the intra-observer variation.*

**Left femoral head projection image**

**
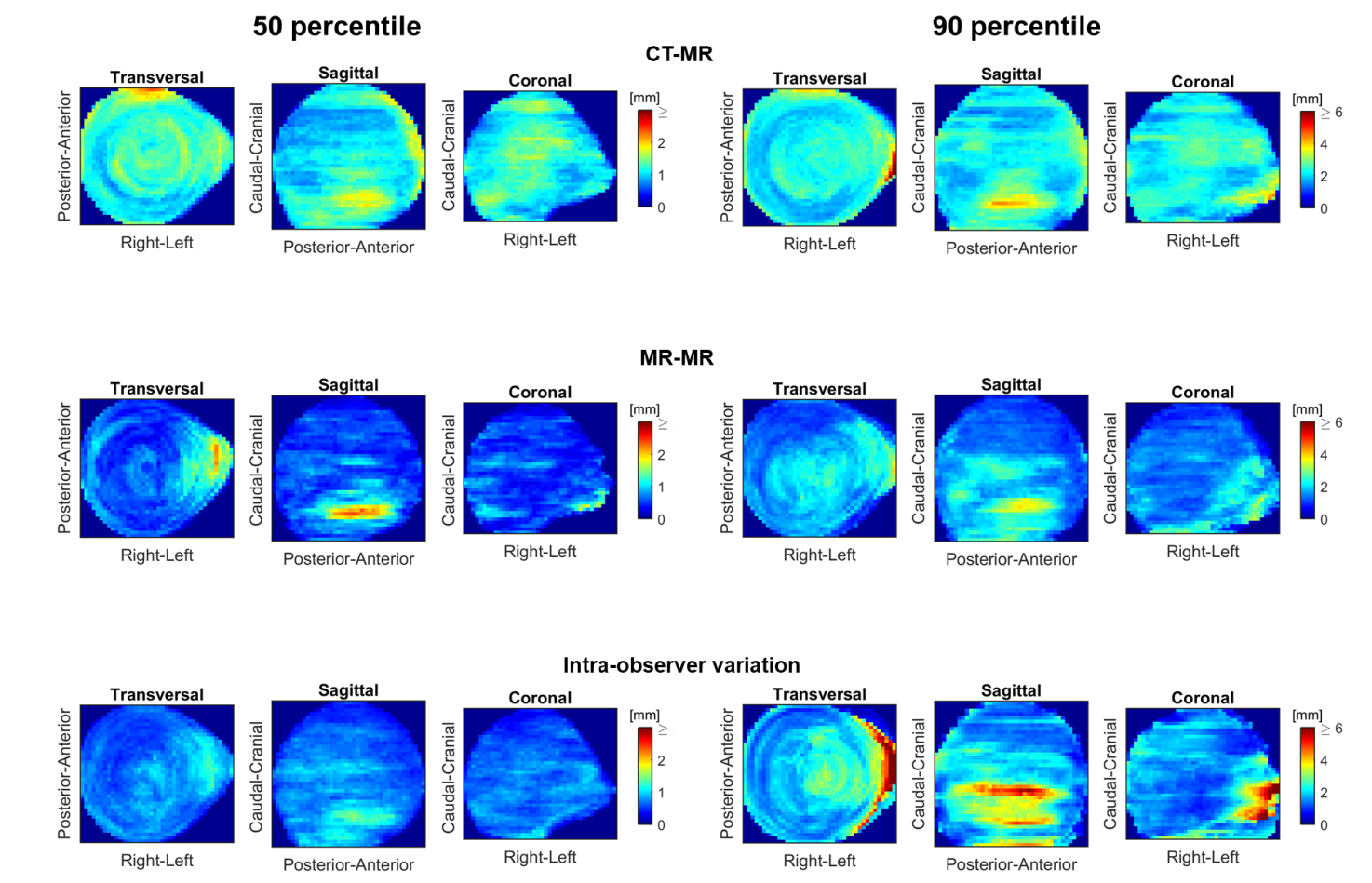
**

*Figure B5.* *50 and 90 percentile surface distance projection images for left femoral head based on CT-MR and MR-MR registrations as well as the intra-observer variation.*

**Penile Bulb projection image**

**
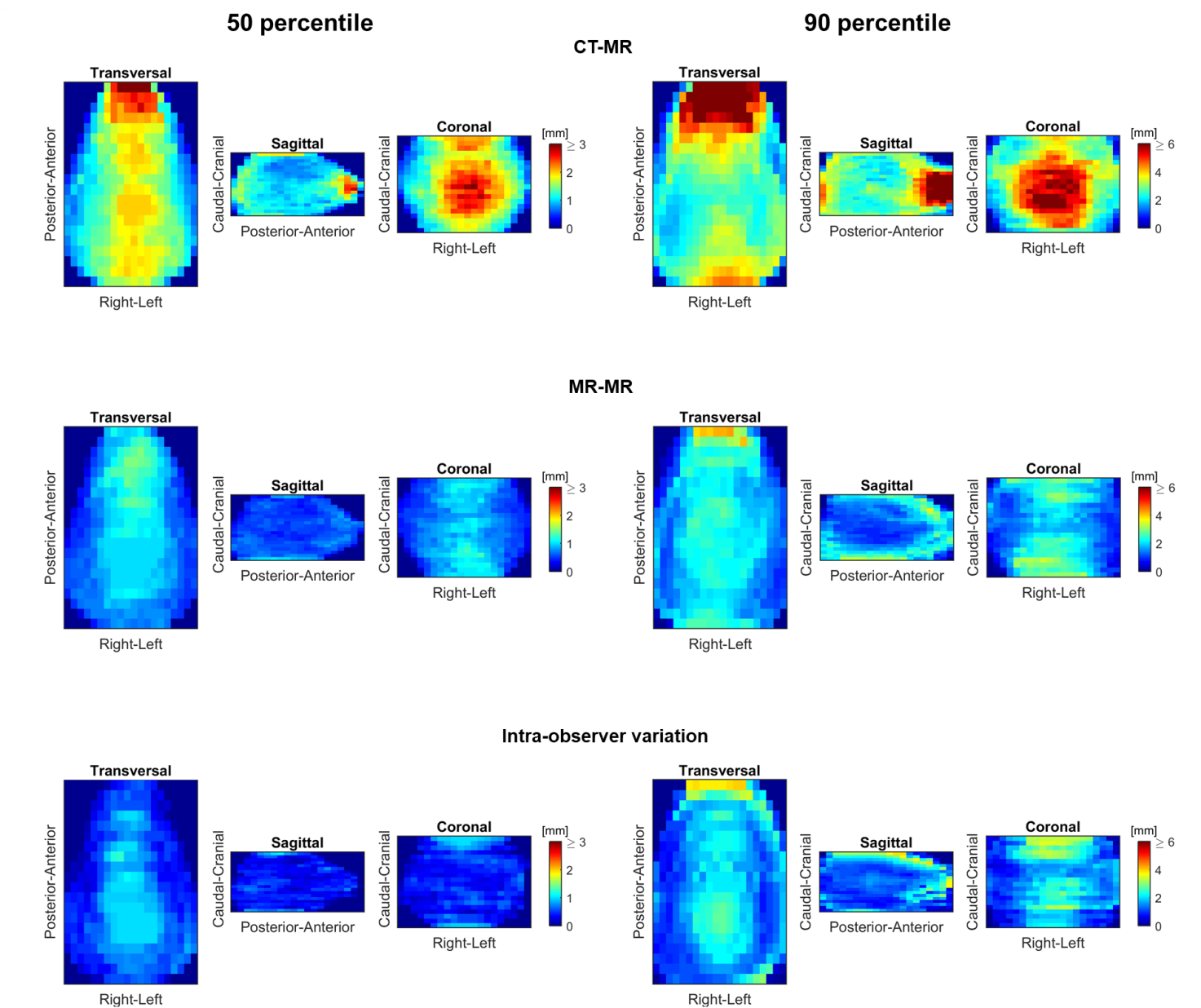
**

*Figure B6.* *50 and 90 percentile surface distance projection images for penile bulb based on CT-MR and MR-MR registrations as well as the intra-observer variation.*
